# *Proceedings B* 2023: the year in review

**DOI:** 10.1098/rspb.2023.2691

**Published:** 2024-01-10

**Authors:** Spencer C. H. Barrett

**Affiliations:** *Editor-in-Chief*

I am pleased to report that during 2023 submissions to *Proceedings B* have increased significantly and we appear to be moving towards a post-COVID recovery. Because of the general interest in the influence of the COVID pandemic on scientific publishing, we have set up a small committee of Editors and they are currently undertaking a detailed analysis of the patterns in submission data during the period 2012–2023 for 25 journals. The dataset has been kindly provided by the publishers of ecology and evolution journals, including Royal Society Publishing. During the year, several other initiatives including double anonymous peer review and the hiring of data editors have been agreed to and will be implemented in the new year. These innovations are discussed more fully below, along with several other exciting new developments at *Proceedings B*. I believe these initiatives will strengthen our reputation and consolidate our position as the world's leading journal in organismal biology.

From 1 January to 31 October 2023 we received 2584 submissions, an increase of 492 in comparison with the same period in 2022. Remarkably of the 2023 submissions, the proportion rejected (75%) was very similar to the two previous years. Up to now, 477 articles have been accepted for publication and of these 279 are open access (OA), a moderate increase over 2022 when 232 were published by this time. Our target for OA content this year is 58.7% of published articles and we are currently at 58.5%. The continuing upward trend is encouraging as our aim is to make the transition to fully open access for *Proceedings B* once we reach 75% open access. Currently, OA content is downloaded about three times more often than subscription content in the journal. Our times (in median days) to first decision (19) have been reduced by 5 days compared with 2022 with times for final decision (72), and times from submission receipt to publication (101) similar to last year. The journal's impact factor dropped from 5.53 to 4.7, a pattern also evident in several other journals publishing similar content. Ecology, evolution and behaviour remain the most popular subject areas for submissions although there have been notable increases for some article types especially those for Evidence Synthesis and Biological Science Practices. *Proceedings B* is currently ranked 16th out of 213 journals in the Journal Citation Reports category for ‘General Agriculture and Biological Sciences'.

We receive submissions from diverse geographical regions, with the largest numbers coming from the USA (544), China (309), UK (270), Germany (131), Australia (130), Canada (106), Japan (96), France (75), Switzerland (61) and Italy (47). These rankings are quite similar to last year, with China maintaining its position having replaced the UK in 2022 as the country with the second most submissions to the journal. We have continued efforts to attract submissions from under-represented regions and one way of doing this is to recruit new Associate Editors from these areas. In this year's round of new recruits, 13% of the 26 new Associate Editors who will start their terms in 2024 are from countries in Asia, Africa and South America. We hope that this will help to stimulate interest in the journal and diversify submissions further.

Review articles are a feature of most issues of *Proceedings B* and provide an outstanding forum for timely overviews of emerging areas and unresolved questions in biology. Reviews editor Innes Cuthill has made particular efforts to increase the range of topics covered by reviews and during the year we have received a steady flow of submissions. To date we have received more Review proposals this year (to the end of October) (74) compared to 2022 (61), of which 42 articles have been submitted with 11 published so far. Those interested in writing a review for *Proceedings B* should download the review proposal form from our website https://royalsocietypublishing.org/rspb/reviews and submit to the review's editor for consideration.

Our biannual review [1] by the *Canadian Society for Ecology and Evolution* President—Stephen Heard (University of New Brunswick) and co-author Julia Mlynarek (Insectarium de Montreal)—is an intriguing article titled ‘Naming the menagerie: creativity, culture and consequences in the formation of scientific names' https://doi.org/10.1098/rspb.2023.1970 [2].

The authors argue that because the nomenclatural codes that govern the naming of species have relatively few constraints, giving new species a Latin binomial is one of the most creative processes in science. They begin their review with a brief history of nomenclature and species naming, including a discussion of the etymology of binomials and the most common kinds found in various taxa. For example, in the four taxa that they examine in detail morphological attributes are most commonly used, although in some groups this practice is declining and the geographical location of the species being named has increased. Other topics covered include the use of languages other than Latin and Greek in species naming and the increase and often controversial use of eponymous names (where a new species is named after a person). An especially interesting feature of the article concerns the consequences of naming for the funding of research and conservation, and also how naming can increase public perception of science and even the direction of research work. The review serves as an excellent reminder that culturally influenced decisions and the social context in which taxonomists work can influence the conduct of science itself.

The annual Darwin review in *Proceedings B*, ‘The evolution of virulence in human pathogens' [2] by Sunetra Gupta (Oxford University) should shortly be published. All pathogens cause some level of harm to their hosts. If this occurs while the pathogen is transmitting, it can negatively affect the fitness of the pathogen by shortening the duration over which transmission occurs. However, many of the factors that increase virulence such as harm caused to the host also promote transmission, driving the pathogen population towards an optimal state of intermediate virulence. In her article, Gupta provides a conceptual framework for the evolution of pathogen virulence and shows that a wider spectrum of virulence may be maintained among pathogen populations which are structured into multiple discrete strains through direct resource and immune-mediated competition. Topics that are covered include the evolution of virulence under complete cross-immunity, the role of immune evasion and host resistance, unstable strain dynamics and the effects of pharmaceutical interventions. The major take home message of the review is that the various evolutionary outcomes, and the effects of medical and public health interventions, are best understood within a framework that recognizes the complex relationship between transmission and virulence in the context of the antigenic diversity of pathogen populations.

At our board meeting in May, we discussed the idea of extending the journal's review portfolio beyond the ‘Darwin Review', which was introduced in 2012. After discussion among Editors we decided to introduce in 2024 the ‘Georgina Mace Review', which will annually showcase current topics in conservation and biodiversity science. The review should be of general interest to our readership and to policy makers and those tasked with the stewardship of natural resources. We named the new review after the late Prof. Georgina Mace FRS for a variety of reasons foremost because she had global recognition for her tireless efforts in promoting the conservation of biodiversity. Her work on the threats posed by environmental change on biodiversity are topics that the Royal Society is currently promoting and having a named review in her honour on the topic of conservation biology aligns well with the goals of the society. Prof. Mace was also a Fellow of the Royal Society and the first female editor of a Royal Society journal (*Philosophical Transactions B*). Prof. Mace's family are delighted with this decision and we are pleased that Eleanor Milner-Gulland, Director of the Interdisciplinary Centre for Conservation Science at the University of Oxford, has agreed to write the inaugural review.

Special Features in *Proceedings B* continue to be a popular forum for in-depth coverage of a single theme through a collection of a dozen or more articles comprised of a mix of both research papers and synthetic reviews. In 2023 our Special Feature is: ‘The resolution of evolutionary conflicts within species' edited by J. Arvid Ågren (Case Western Reserve University), Göran Arnqvist (Uppsala University) and Locke Rowe (University of Toronto). Evolutionary conflicts occur at all levels of biological organization and are a fundamental component of life. They range from conflicts between genetic elements and cells, conflicts between the sexes, to conflicts between competing individuals within populations. The existence of well-functioning genomes, complex multi-cellular individuals, and societies suggest that ecological and evolutionary processes exist that may function in resolving or mitigating conflicts. The Special Feature focuses on the major sources of evolutionary conflicts within species, the mechanisms that limit conflict at each level, and current evidence in support of conflict resolution. A central goal of the Special Feature is that it aims to be synthetic and searches for shared ideas, themes and mechanisms across systems that can contribute to the development of a general evolutionary theory of sexual conflict and its resolution. Several articles for the Special Feature are now online and the entire collection will be published early next year once all papers have gone through review and are accepted.

Several new journal developments were decided at our May editorial board meeting in the hope that they will improve the quality of decision making at the journal in 2024. Recent studies [e.g. 3] of double anonymous (blind) peer review indicate that reviewer bias is significantly reduced when author identities are anonymized and reviewers do not know the authors and their institutions. Starting 1st January, *Proceedings B* will operate with mandatory double anonymous peer review (DAPR—author and reviewer names kept hidden throughout the review process). Editors will still be able to view author names and affiliations but editors and reviewers are still expected to handle the manuscripts confidentially and must not disclose any details to anyone outside of the review process. As with other journals that practice DAPR, it is the responsibility of authors to anonymize their articles and associated data files, and all articles should be submitted so that they are ready for DAPR, with a separate title page and all obviously identifiable references to authors removed from the manuscript, supplementary files and data files submitted to third party repositories. Inadequate anonymization may result in paper being returned to authors. We encourage authors to follow the *Proceedings B* double anonymous peer review ‘Instructions for Authors' when preparing articles for submission—these are available on the journal website—https://royalsocietypublishing.org/rspb/double-anonymous-peer-review.

A second innovation that will be introduced in 2024 involves the use of data editors at *Proceedings B.* Managing and evaluating the quality and accessibility of data, code and statistical analyses in published articles is a growing problem because reviewers and editors often do not scrutinize all data files. But journals need to ensure that data and code supporting the conclusions of an article have been deposited in an appropriate repository, are made freely accessible, and are comprehensible and free from errors. This is an important element of how science is perceived, trusted and used by the public to inform policy [4]. However, there is increasing evidence that many published articles do not meet the data requirements set by journals. At *Proceedings B*, our data sharing and mining policies state that it is a condition of publication that authors make available the data, code and research materials supporting the results in their articles. This practice allows others to verify and build on the work published and serves to promote greater openness in scientific research. Data and code must be made available *on submission* in an external repository or as supplementary material so that editors and reviewers can access this information and confirm that the archive is complete and usable by others. At *Proceedings B* it is not permitted to request that data will be available from the authors upon request; however, exceptions may be made when ethical standards require that data be kept confidential (e.g. with certain kind of human genetic data). If our guidelines are not met, submitted articles will be sent back to the authors for revision.

With the concern by many that research integrity is under threat, and as part of our efforts to ensure that the journal's data policies are adhered to, *Proceedings B* has recruited a group of data editors to join the journal's editorial board. The role of the data editor will be to: (i) Check data statements and the data and code on accepted articles to ensure that all data, code and research materials supporting the results have been deposited in a suitable repository and are fully accessible; (ii) Verify that there are no anomalies in the deposited material that cast doubt on the reliability of the research and its main conclusions; (iii) Be called upon to check and verify datasets in previously published articles where concerns have been raised. To recruit our data editors, we advertised through social media and sought recommendations from our Editors and we obtained 42 applicants. We were especially interested in having a broad range of expertise and experience among the editors because of the diverse subject areas that we publish in *Proceedings B*. I am pleased to say that we have recently recruited eight individuals from diverse countries (Australia, Germany, Netherlands, Sweden, Switzerland, USA and UK) with expertise ranging from statistical analysis and mathematical models, quantitative ecology and big data, environmental sciences, bioinformatics and genomics, comparative and developmental biology and palaeobiology, anthropology and human studies. We recently conducted a video workshop with our data editors, and advisor Robert Montgomerie from *The American Naturalist,* to discuss the role of data editors at *Proceedings B* and the development of standard editorial practices so that their duties are integrated into the journal's work flow and are ready for implementation in 2024.

Another new development this year at *Proceedings B* involves an online platform that hosts seminars with questions and answers (Q&A) on recent articles published in the journal. This ecology and evolution channel—Cassyni—that has been set up Royal Society Publishing is an excellent way of increasing the visibility of research that we publish. Speakers are given a one-hour time slot to discuss their highlighted papers with an Associate Editor hosting the seminar, and a Q&A session at the end. Seminars are citable with a DOI and are available online and free for anyone to view. Viewership numbers are collected by our marketing team and to date *Proceedings B* has hosted six seminars the first of which (see here, seminar) was presented by Diego Vazquez (National University of Cuyo, Argentina) and hosted by Associate Editor Marcel Dorken (Trent University, Canada) had 45 people view the seminar live, with more online views since. Several more seminars are planned for the coming months.

A pressing issue that all scientific journals have been recently grappling with concerns the use of Artficial Intelligence (AI) and AI-assisted technologies in the submissions they receive. Indeed, this year we received the first such submission to *Proceedings B*. Because AI will undoubtedly play an increasing role in scientific research, and also in the communication of results via publication, it was critically important that Royal Society Publishing develop a set of policy guidelines for authors. This has now been done and is available on the Royal Society website https://royalsociety.org/journals/ethics-policies/openness/ in a section specifically on AI. Briefly, all authors must disclose in their manuscripts the use of AI, and a statement will be required in the published work. The statement should provide details of which elements of the work were generated by AI and AI-assisted technologies. Editors and reviewers will judge if the use of AI is appropriate. Where used in the writing process, these technologies should only be used to improve readability and language of the work. Use of AI in language editing must be declared. AI may be used as a ‘search engine' e.g. to aid identification of suitable code or statistical techniques, but should not replace key researcher tasks such as producing scientific insights, analysing and interpreting data or drawing scientific conclusions. And finally, authors should not list AI and AI-assisted technologies as an author or co-author of a paper and submissions must be an accurate representation of the novel intellectual contributions of authors and not primarily the result of the generative capabilities of AI-assisted technologies. Published articles in which the use of such technologies was not initially disclosed and was subsequently discovered may be retracted.

To conclude, I would like to congratulate Senior Editors Prof. John Hutchinson (Royal Veterinary College, London) and Prof. Loeske Kruuk (University of Edinburgh) who were both elected in 2023 to the fellowship of the Royal Society of London in recognition of their novel contributions to the fields of evolutionary biomechanics and quantitative genetics, respectively. I also extend thanks to the Senior and Associate Editors of *Proceedings B* for all their hard work in handling the large number of submissions that we receive. For those completing their terms and leaving in 2024, I hope that your time with *Proceedings B* has been generally enjoyable, despite the inevitable ‘editorial headaches' and that you have made new contacts and expanded your networks. I would also like to thank our editorial team in London, consisting of Editorial Coordinators Jennifer Kren and Callum Shoosmith, Teagan Meadows (Production Coordinator) and Amie Mustill (Production Manager) for their attention to detail and conscientious work in making sure that *Proceedings B* runs efficiently and on time. I also thank Sophie Young, who during 2023 took up a work experience at Royal Society Publishing and assisted us with handling papers at Scholar One. I would especially like to thank the Publishing Editor of *Proceedings B,* Shalene Singh-Shepherd for her continuing dedication to the journal and sage advice. I am especially grateful to Shalene for spearheading our new initiative on submission patterns to journals in organismal biology pre- and post-COVID, and in establishing valuable working relationships with the publishers supplying data to our study. Finally, I would like to thank Stuart Taylor who this year retired as Publishing Director at Royal Society Publishing. Stuart has been a wonderful source of calm, considered and expert advice during challenging periods, especially those involving the misconduct investigations that we have mounted in recent years. I wish Stuart all the best in his well-earned retirement and I look forward to working with the new Publishing Director Rod Cookson. From all of us at *Proceedings B*, we wish you all a peaceful and healthy 2024 and we look forward to another productive year at the journal.
